# Using mHealth to Improve Usage of Antenatal Care, Postnatal Care, and Immunization: A Systematic Review of the Literature

**DOI:** 10.1155/2015/153402

**Published:** 2015-08-25

**Authors:** Jessica L. Watterson, Julia Walsh, Isheeta Madeka

**Affiliations:** ^1^Private Practice in Maternal and Child Health, Berkeley, CA 94704, USA; ^2^207L University Hall, University of California, Berkeley, CA 94704, USA; ^3^University of California, Berkeley, CA 94704, USA

## Abstract

Mobile health (mHealth) technologies have been implemented in many low- and middle-income countries to address challenges in maternal and child health. Many of these technologies attempt to influence patients', caretakers', or health workers' behavior. The purpose of this study was to conduct a systematic review of the literature to determine what evidence exists for the effectiveness of mHealth tools to increase the coverage and use of antenatal care (ANC), postnatal care (PNC), and childhood immunizations through behavior change in low- and middle-income countries. The full text of 53 articles was reviewed and 10 articles were identified that met all inclusion criteria. The majority of studies used text or voice message reminders to influence patient behavior change (80%, *n* = 8) and most were conducted in African countries (80%, *n* = 8). All studies showed at least some evidence of effectiveness at changing behavior to improve antenatal care attendance, postnatal care attendance, or childhood immunization rates. However, many of the studies were observational and further rigorous evaluation of mHealth programs is needed in a broader variety of settings.

## 1. Introduction

Despite ongoing efforts to improve maternal and child health in developing countries, mortality rates remain much higher than in developed countries. Women in developing regions face a lifetime risk of maternal death of 1 in 160, as compared with 1 in 3700 for women living in developed regions [1]. These inequalities are driven by many causes, one of which is limited access to preventive services. For example, in low- and middle-income countries, only about 52% of pregnant women receive the World Health Organization- (WHO-) recommended minimum of four antenatal visits [2]. The postnatal period is also critical to the health of a mother and newborn, as the majority of postnatal maternal deaths happen during the first week after birth [3]. However, a recent analysis of Demographic and Health Surveys for 23 African countries found that, of the two-thirds of women giving birth at home, only 13% received a postnatal check-up within two days [3]. Immunization is another critical preventive service that can save the lives of many infants and children. Despite being one of the most cost-effective tools for saving lives, nearly one in five children globally did not receive their full package of immunizations in 2012 and 1.5 million children under the age of 5 died from vaccine-preventable diseases in 2008 [4, 5]. Antenatal care (ANC), postnatal care (PNC), and childhood immunization make up an important package of preventive services that can improve maternal and child health. Families tend to use medical services when someone is ill but frequently omit these beneficial preventive services that are essential to improve health.

The field of mHealth, or mobile health, has been proposed as a potential solution to many of the challenges that developing countries face, including workforce shortages, lack of health information, limited training for health workers, and difficulty tracking patients. mHealth projects have been implemented all over the world, using mobile phones for record keeping, data collection, or patient communication [6]. Further, mHealth tools have been used to promote behavior change in health workers and/or patients. For example, text message reminders have been shown to increase care-seeking behavior or medication adherence in some patients and mobile data collection and communication tools for health workers have improved follow-up of patients and data reporting [7–9].

Though there are relatively few thorough evaluations of mHealth programs [6], some published studies do exist. Given that mHealth tools have shown some promise for behavior change more broadly, there is potential for this field to improve essential preventive maternal and child health services as well. Based on the existing evidence in peer-reviewed publications, this literature review aims to determine the effectiveness of mHealth tools to increase the coverage and use of antenatal care, postnatal care, and childhood immunizations through behavior change in low- and middle-income countries.

## 2. Materials and Methods

### 2.1. Information Sources

This literature review was conducted through a keyword search of the following databases to identify relevant peer-reviewed articles: Google Scholar, PubMed, Embase, PsycINFO, and EBSCO Host. Keywords used in these searches included* mHealth*,* mobile health*,* mobile phone*,* reminder*,* recall*,* mobile medical records*,* antenatal care*,* postnatal care*, and* immunization*.

### 2.2. Inclusion Criteria

In order to be included in the review, the article had to meet the following inclusion criteria:

- study was evaluating an mHealth intervention targeted at increasing antenatal care attendance, postnatal care attendance, or childhood immunization rates through behavior change;
- study was implemented in a low- or middle-income country;
- study included measurement of process, behavior change, health, or quality of care outcomes (i.e., studies were excluded that only evaluated willingness of participants to receive an mHealth intervention, without implementing it);
- study was a peer-reviewed article;
- study was available in English;
- study was published between January 1, 2000 and November 20, 2014.

These criteria were selected to ensure that the included studies examined outcomes of existing mHealth interventions, not exploratory studies or protocols that have not been implemented yet. Low-, middle-, or high-income status for countries was determined using the World Bank's 2014 classification, which is based on estimates of the gross national income per capita for the previous year [10]. In addition, the inclusion of only peer-reviewed articles helped to ensure that higher quality studies were examined. Though there have been well-designed studies using mHealth for behavior change to support maternal and child health in high-income countries, these studies were excluded due to the resource disparities between high-income countries and others. Issues with prevalence of mobile phones and consistency of power and internet access are shared across many low- and middle-income countries and therefore these studies are more comparable than those conducted in high-income countries. No keywords for “low- or middle-income countries” were used in the searches, as these keywords might have excluded relevant results if the study was not specifically labeled as such. Instead, the authors screened manually for this criterion. The review was limited to studies available in English, though this is a limitation of this review, and future reviews should include additional languages, if feasible. Finally, studies only included those that were published after 2000, as mobile technologies were not widely available, especially in low- and middle-income countries, prior to that time.

### 2.3. Study Selection and Data Collection

The database searches were undertaken by two researchers (Jessica L. Watterson and Isheeta Madeka) between November 10, 2014 and January 18, 2015. Subsequent review of results was undertaken by one researcher (Jessica L. Watterson). The resulting articles were first screened by title, then by abstract, and finally by full text to progressively eliminate articles not meeting the inclusion criteria. Many systematic reviews of mHealth research were identified in the results (*n* = 26), so the included articles and reference lists of these reviews were all examined to ensure an exhaustive search. Finally, the references of all included articles were reviewed as well.

**Figure 1:**
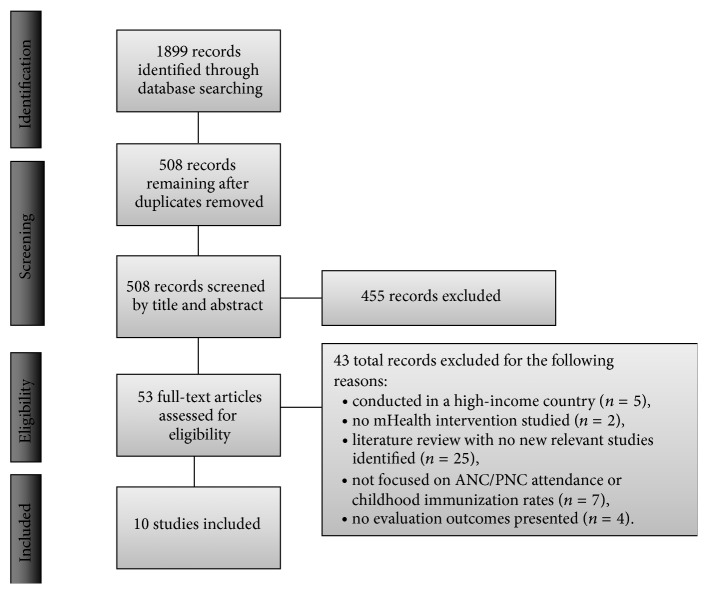
PRISMA flow diagram [26].

The results of study screening and selection are illustrated in Figure 1. The database searches identified 1,899 articles initially. After removing duplicates, 508 records remained. Each of these records was screened by title and abstract (if necessary), and 455 records were excluded after this preliminary review. The full text of the remaining 53 articles was reviewed to determine if they met the inclusion criteria. 43 of the articles were excluded and the reasons for exclusion included study being conducted in a high-income country (*n* = 5); not studying antenatal care attendance, postnatal care attendance, or childhood immunization rates (*n* = 7); not studying an mHealth intervention (*n* = 2); or only providing program descriptions or a protocol but no evaluation data (*n* = 4). The other 25 articles that were excluded were mHealth literature reviews that did not identify any new articles for review. One article outlined a protocol for a study that will be very relevant once complete; however it was nevertheless excluded because no evaluation data is published yet [11].

### 2.4. Quality Assessment

Risk of bias was assessed for all included randomized controlled trials (RCTs) (*n* = 2) using the Cochrane Risk of Bias Assessment Tool [12]. This tool was introduced in 2008 by the Cochrane Collaboration and can be used to assess risk of bias in a study by evaluating a study's allocation sequence generation (randomization), allocation concealment, blinding, incomplete data, selective reporting, and other potential threats to the study's validity. The quality of the observational studies (*n* = 8) was assessed using the Newcastle-Ottawa Quality Assessment Scale [13]. This tool was developed by the universities of Newcastle, Australia, and Ottawa, Canada, and assessed the quality of nonrandomized studies by evaluating potential sources of bias in the selection and comparability of participants, the assessment of outcomes, and the duration and adequacy of follow-up. Scores are awarded out of 9 possible points, with higher scores indicating higher study quality.

### 2.5. Synthesis of Results

The primary author (Jessica L. Watterson) extracted information from included articles for tabulation in an Excel spreadsheet. The information extracted included type of study, summary conclusions, methods used, intervention studied, health issue(s) studied, outcomes measured, sample size, intervention frequency, effectiveness of intervention, study quality, study location, clinical characteristics/setting, mHealth tools used, and project name (if any).

## 3. Results

Most articles examined process and behavior change outcomes and made recommendations for future mHealth programs and suggested further research. The study characteristics and key outcomes for each included article are outlined in Table 1.

### 3.1. Characteristics of Studies

In total, ten articles satisfied the inclusion criteria. Of these, two studies were RCTs [14, 15] and the other eight were observational studies [16–23]. Four of the observational studies attempted to limit sources of bias (though not as rigorously as the RCTs) by using a historic control group [16, 17] or nonrandomized control group [19] or measuring outcomes before and after implementation of the mHealth intervention [18]. The remaining four observational studies did not use a control group [18–21], and as such the outcomes of these studies are less reliable.

Seven (70%) of the articles studied antenatal care attendance [14, 15, 17–20, 22]; two (20%) studied postnatal care attendance [16, 20]; and four (40%) studied childhood immunization rates [18, 20, 21, 23]. Eight (80%) of the studies used an mHealth intervention that sent reminders to seek care directly to patients [14–18, 20, 21, 23] and five (50%) sent educational messages to patients [14, 15, 17, 19, 20]. Three (30%) studies sent reminders to health workers to follow up with patients [18, 21, 22] and three (30%) studies used an mHealth tool to improve patient records or identification [18, 21, 22]. The frequency of these interventions varied widely; educational messages were sent on schedules ranging from daily [19] to twice per month [15]. Some studies specified that appointment reminders were sent a few days in advance of a scheduled appointment [16, 18, 23] and others did not specify how far in advance patients or health workers were reminded.

Eight (80%) of the studies were conducted in Africa [14–16, 19–23] and two (20%) were conducted in Asia [17, 18]. All included studies were published between 2010 and 2014, suggesting that the inclusion criterion of studies published after 2000 was sufficiently conservative and that it is unlikely that any relevant articles were missed from earlier publication dates. One study is taken from a literature review published in English on mHealth tools in China [17]; however, the original study was published in Chinese. Therefore, the information available on this study is less complete than that provided for the studies where the primary publication was included.

### 3.2. Findings by Intervention

All studies showed some evidence that the mHealth intervention implemented had a positive impact on patient or health worker behavior. However, the quality of the studies varied and some of these outcomes cannot be conclusively attributed to the mHealth intervention that was implemented from these studies alone.

#### 3.2.1. Antenatal Care Attendance

Two of the seven studies examining antenatal care attendance were RCTs [14, 15]. Both studies used text message reminders and education for pregnant women and one also provided the women with mobile-phone vouchers to contact their health worker, if needed [15]. Both studies found a statistically significant increase of over 10% in the proportion of women receiving at least four antenatal care visits between the intervention and control groups. Another study examined antenatal care attendance before and after implementation of an mHealth application for improved patient records and automated appointment reminders; this study similarly found a statistically significant improvement in on-time antenatal care attendance following implementation [18]. A study conducted in China sent text message reminders for antenatal care and health advice to an intervention group and found a statistically significant increase in antenatal care attendance, compared to a historic control group. The remaining studies examining antenatal care attendance found some self-reported behavior change from both patients and health workers [19, 20, 22].

#### 3.2.2. Postnatal Care Attendance

One study examining postnatal care attendance used a historic control group from the previous 6 months in the same hospital and found that the intervention group, receiving text message appointment reminders, were 50% less likely to fail to attend their appointment (*P* = 0.002) [16]. Another study found that women self-reported intended or actual behavior change, including increased attendance to postnatal care, after receiving voice or SMS messages with education and reminders [20].

#### 3.2.3. Childhood Immunization

A study examining childhood immunization found a statistically significant increase (from 34.5% to 44.2%, *P* < 0.001) in the proportion of children receiving on-time vaccination after implementation of a mobile application for improved patient records and automated text message appointment reminders [18]. Another study found that mothers reported being influenced by a text message reminder (which were also tied to a conditional cash transfer, if child was vaccinated on time) to bring their child for immunization [23]. In one study, after receiving SMS or voice reminders and education, mothers self-reported intended or actual behavior change, including bringing their child for vaccines [20]. Finally, a study using an mHealth application to improve records and to send reminders during a mass vaccination campaign found that 92% of children visited at home following the campaign had received the measles vaccine [21].

### 3.3. Findings on Cost

Two of the studies included information on the cost of their mHealth interventions. Adanikin et al. reported a total cost of only US$21.12 to send 2252 SMS reminders for postnatal care during the six-month study in Nigeria [16]. Ngabo et al. cited initial investment cost as being considerable, largely due to the fact that they provided all community health workers in Rwanda with a mobile phone to “boost engagement and motivation of CHWs.” However, ongoing costs were lowered by Ministry of Health negotiations with the private sector, reducing SMS costs from US$0.05 to US$0.005 per message [22].

## 4. Discussion

Though all included studies showed some evidence that mHealth tools can be effective in changing patient and health worker behavior to increase antenatal care attendance, postnatal care attendance, and childhood immunization rates, the quality of the evidence varied widely.

The strongest evidence exists for text message reminders and education delivered to pregnant women's mobile phones. The two RCTs that examined this intervention both found evidence of statistically significant increases in antenatal care attendance in their intervention groups, relative to their control groups [14, 15]. There is also some suggestion from the results that this intervention may also be effective when applied to the other health issues studied, such as postnatal care attendance and childhood immunization. Though no RCTs studied these health issues, two observational studies of high quality found evidence of effectiveness. Adanikin et al. found that intervention group receiving text message reminders for postnatal care were 50% less likely to fail to attend their appointments than a historic control group from the previous six months (*P* = 0.002) [16]. Kaewkungwal et al. found that after implementation of a smartphone application that supported record keeping and generated text message reminders for health workers and mothers, there was a 15% increase in women attending ANC on time (*P* < 0.001) and a 10% increase in children receiving on-time immunizations (*P* < 0.001) [18].

Beyond these findings, much of the evidence is based on self-reported behavior change from health workers and patients, which is not sufficiently reliable to draw any strong conclusions on the effectiveness of mHealth interventions [20, 22, 23]. Some other observational studies demonstrated good results of their programs, such as 92% confirmed coverage in a measles vaccine campaign [21]; however, it is impossible to determine which factors influenced the campaign's success and whether it was due to the use of an mHealth intervention or one of the other program components. In addition, several studies combined multiple mHealth interventions (e.g., text message reminders and conditional cash transfers via mobile phone [23]), making it impossible to determine to what degree each intervention influenced the resulting behavior change.

As a result of these methodological limitations and the small number of studies meeting the inclusion criteria, further randomized controlled trials are needed to evaluate the effectiveness of mHealth tools for antenatal care, postnatal care, and childhood immunizations. By employing a multiarm or factorial design, researchers may be able to better ascertain which components of mHealth interventions are most effective.

It is also worth noting that many of the mHealth tools studied focused on a single period of time on the maternal, neonatal, and child health (MNCH) continuum. For example, one study focused only on postnatal care, while five others focused only on antenatal care. Given the importance of continued follow-up of families during pregnancy, delivery, postnatal periods, and early childhood, it would be advisable for future mHealth interventions to consider expanding their tools to include more key events along the MNCH continuum [23]. Finally, the majority of these studies were conducted in Africa, suggesting that there is a need for future study of mHealth tools in broader contexts, including Asia and the Pacific, Central and South America, the Caribbean, and other regions.

This literature review has provided us with the key knowledge that there is some existing evidence of the effectiveness of text message reminders for antenatal care, postnatal care, and immunizations and it has also helped to identify that this is an area where further research is needed. Given the limited, but largely positive, results of this literature review, researchers and public health practitioners should continue to implement mHealth tools for antenatal care attendance, postnatal care attendance, and childhood immunization. However, careful evaluation and further research are still needed to better determine how effective these tools are and in which settings.

## 5. Conclusions

Based on a systematic review of the literature, there is some evidence that mHealth tools may present an opportunity to influence behavior change and ensure that women and children in low-income countries are accessing prevention services, including antenatal care, postnatal care, and immunizations. Though mHealth programs have been implemented in low- and middle-income countries all over the world [24], there are few peer-reviewed studies and the majority of evaluations relating to maternal and child health have been conducted in Africa. Therefore, greater emphasis needs to be put on the evaluating mHealth tools and disseminating results to inform program design and policy making. In addition, many existing interventions focus on only one component of maternal and child health preventive services, rather than on design of an integrated system that follows women and children through the maternal, neonatal, and child health continuum [25]. The field of mHealth should continue to be supported and studied as it shows promise of improving the lives of women and children in low- and middle-income countries.

## Figures and Tables

**Table 1:** Summary of included articles on mHealth interventions to increase use of antenatal care, postnatal care, and childhood immunization, classified by methods used.

First author, year	Title	Health issue(s) studied	Intervention studied and tools used	Intervention frequency	Key study outcomes	Methods used	Sample size	Study location	Study quality^1^
Randomized controlled trials (RCTs)									

Fedha, 2014 [14]	“Impact of Mobile Telephone on Maternal Health Service Care: A Case of Njoro Division”	Antenatal care attendance	Text message reminders and educational messages for mother delivered to mobile phone. No specific mHealth tools mentioned	Appointment reminders every two weeks. Frequency of educational messages not specified	7.4% of women receiving SMS had less than 4 antenatal visits while 18.6% of those not receiving SMS had less than 4 visits (*P* = 0.002)	Clinic attendance and antenatal service uptake compared for intervention and control groups	Intervention group: 191 Control group: 206 Total: 397	Health facilities in Kenya	RCT with low risk of bias

Lund, 2014 [15]	“Mobile Phones Improve Antenatal Care Attendance in Zanzibar: A Cluster Randomized Controlled Trial”	Antenatal care attendance	Text message reminders and educational messages for mother delivered to mobile phone and mobile vouchers to contact health workers. Tools used: custom Wired Mothers software	Two messages per month before gestational week 36 and two messages per week after week 36	44% of women in the intervention group received the recommended four or more antenatal visits, compared with 31% in the control group. The odds for receiving four or more antenatal care visits were 2.39 (1.03–5.55) for women benefitting from the mobile phone intervention. 59% of intervention women stated that received text messages influenced the number of times they attended antenatal care	Clinic attendance was compared for cluster randomized intervention and control groups	Intervention group: 1311 Control group: 1239 Total: 2550	Urban and rural healthcare facilities in Zanzibar	RCT with low risk of bias

Studies with nonrandomized control group or before/after design									

Adanikin, 2014 [16]	“Role of Reminder by Text Message in Enhancing Postnatal Clinic Attendance”	Postnatal care attendance	Text message reminders for mother delivered to mobile phone. No specific mHealth tools mentioned	Two messages sent for each appointment: two weeks prior and 5 days prior	Patients who received an SMS reminder were 50% less likely to fail to attend (FTA) their postnatal appointment (relative risk of FTA 0.50; 95% CI, 0.32–0.77; *P* = 0.002)	Clinic attendance compared for intervention group and historic control group (from previous 6 months)	Intervention group: 1126 Control group: 971 Total: 2097	Teaching hospital in Nigeria	7/9

Fang and Li, 2010 [27] from Corpman, 2013 [17]	“Mobile Health in China: A Review of Research and Programs in Medical Care, Health Education, and Public Health”	Antenatal care attendance	Text message appointment reminders and antenatal health advice. No specific mHealth tools mentioned	Four appointment reminders per pregnancy. Frequency of health advice not specified	The intervention group received 5.7 ± 1.8 antenatal visits, compared to 3.2 ± 1.1 antenatal visits in the control group (*P* < 0.01)	Clinic attendance compared for intervention group and historic control group (from previous year).	Intervention group: 609 Control group: 637 Total: 1246	China	Unable to determine as not all info. on study design is available in English

Kaewkungwal, 2010 [18]	“Application of Smart Phone in “Better Border Healthcare Program”: A Module for Mother and Child Care”	Antenatal care attendance and childhood immunization (EPI)	Smartphone application used by health workers to update antenatal and immunization status when outside clinic and SMS reminders for both health workers and mothers. Tools used: custom Mother and Child Care Module (MCCM)	Appointment reminders a few days prior to scheduled appointment	58.68% of pregnant women came to ANC on time after implementation as compared to 43.79% before (*P* < 0.001). After adjusting for personal characteristics, sending appointment message increased odds of on-time visit by 2.97 (1.60–5.54). 44.22% of children received scheduled vaccines on time after implementation as compared to 34.49% before (*P* < 0.001). After adjusting for personal characteristics, follow-up cases and updating immunization data on cell phones increased odds of on-time EPI by 2.04 (1.66–2.52). Sending appointment reminder increased odds of on-time EPI by 1.48 (1.09–2.03)	Clinic attendance for ANC and EPI were compared before and after MCCM implementation	ANC group: 280 EPI group: 544	Rural border area in Thailand, near Myanmar	8/9

Lau, 2014 [19]	“Antenatal Health Promotion via Short Message Service at a Midwife Obstetrics Unit in South Africa: A Mixed Methods Study”	Antenatal care attendance	Text messages with antenatal health information. No specific mHealth tools mentioned	Varied from three messages per week to daily messages	92% of participants in the intervention group reported not missing more than two antenatal visits. A focus group of intervention participants reported that they had improved health related behaviors, including attending the clinic regularly, as a result of the text messages. No statistically significant difference in knowledge was seen between the intervention and control groups at the exit interview	Baseline questionnaire and exit interview were administered to convenience-sampled intervention and control groups to assess knowledge of antenatal health and clinic procedures. A focus group was conducted with a further convenience sample of the intervention group	Intervention group: 102 but 45 were lost to follow-up Control group: 104 but 43 were lost to follow-up Total: 206 recruited, 118 included in analysis	Urban primary care facility in Cape Town, South Africa	4/9

Studies with no control group									

Crawford, 2014 [20]	“SMS versus Voice Messaging to Deliver MNCH Communication in Rural Malawi: Assessment of Delivery Success and User Experience”	Antenatal care attendance, postnatal care attendance, and childhood immunization	Text (SMS) or voice message reminders and educational messages for mother delivered to mobile phone or retrieved by calling a toll-free hotline. Tools used: Village-Reach custom application (SMS) and INTELLIVR software (voice messages)	Once (voice) or twice (SMS) per week	91% of SMS enrollees surveyed reported that they had already changed or intended to change their behavior based on the messages, including attending more ANC/PNC or bringing their child for vaccines. SMS enrollees were significantly more likely to report intended or actual behavior change than voice enrollees	Phone based surveys of participants. Participants in the pushed SMS and pushed voice groups were randomly sampled but participants in the retrieved voice group were convenience sampled	Pushed SMS: 96 Pushed voice: 30 Retrieved voice: 140 Total: 266	Rural health centers in Malawi	2/9

Mbabazi, 2014 [21]	“Innovations in Communication Technologies for Measles Supplemental Immunization Activities: Lessons from Kenya Measles Vaccination Campaign, November 2012”	Childhood immunization	Smartphone application used by volunteers to update immunization records when canvassing door-to-door and to provide text message and phone call reminders to caretakers. Tools used: EpiSurveyor	Varied/as needed	In precampaign house-to-house visits, 25% of households had no plans to bring their children for the measles supplemental dose if they had not been contacted by the volunteers. Of the children found in the postcampaign house visits, 96% reported to have received a measles supplemental immunization dose, although only 92% had confirmation (finger mark) of vaccination	Precampaign household canvassing and data collection for entire target population, followed by postcampaign verification of vaccine coverage	Precampaign: 164,643 households with 161,695 children Postcampaign: 17,627 households with 17,993 children	Urban areas in Kenya	5/9

Ngabo, 2012 [22]	“Designing and Implementing an Innovative SMS-based Alert System (RapidSMS-MCH) to Monitor Pregnancy and Reduce Maternal and Child Deaths in Rwanda”	Antenatal care attendance	Electronic registration of pregnant women through text messages by community health workers (CHWs) and reminder text messages for antenatal care sent to CHWs' mobile phones. Tools used: customized version of RapidSMS	As needed for upcoming antenatal visits and estimated delivery date	81% of the estimated annual pregnancies in the district were registered in the system. Reporting compliance among CHWs was 100%. CHWs reported being more proactive in finding new pregnant women and following up registered pregnant women as a result of reminders forwarded to their mobile phones	Reporting compliance, system usage patterns, and error rates were monitored and feedback sessions were held with CHWs	CHWs: 432	Rural district of Rwanda	N/A, only process outcomes were studied

Wakadha, 2013 [23]	“The Feasibility of Using Mobile-Phone Based SMS Reminders and Conditional Cash Transfers to Improve Timely Immunization in Rural Kenya”	Childhood immunization	Text message reminders for mother delivered to mobile phone and free airtime or mobile cash transfers for mothers that brought child in on time. Tools used: customized version of RapidSMS and mPESA	Three days before vaccine due date and on due date	91% of mothers reported that the SMS reminders influenced their decision to come in for vaccination	Enrolled mothers were randomized to receive either mMoney or airtime for on-time vaccinations. Questionnaires were administered in home follow-up visits	mMoney group: 48 Airtime group: 24 Total: 72	Rural district of Kenya	4/9

^1^Quality score assigned using the Cochrane Risk of Bias Assessment Tool (for RCTs) or the Newcastle-Ottawa Quality Assessment Scale (for observational studies). For RCTs, a low risk of bias is the best possible score and for observational studies the highest possible score is 9. Please see Section 2 for more details.
